# Supplementary material for: Anti-Influenza Activity of 6BIGOE: Improved Pharmacological Profile After Encapsulation in PLGA Nanoparticles
Source: Int J Mol Sci. 2025 Apr 29;26(9):4235. doi: 10.3390/ijms26094235 (PMC12071637; doi:10.3390/ijms26094235)
Supplement: Supplementary file 1 [file ijms-26-04235-s001.zip › ijms-3564052-supplementary.pdf]

Supplementary Materials

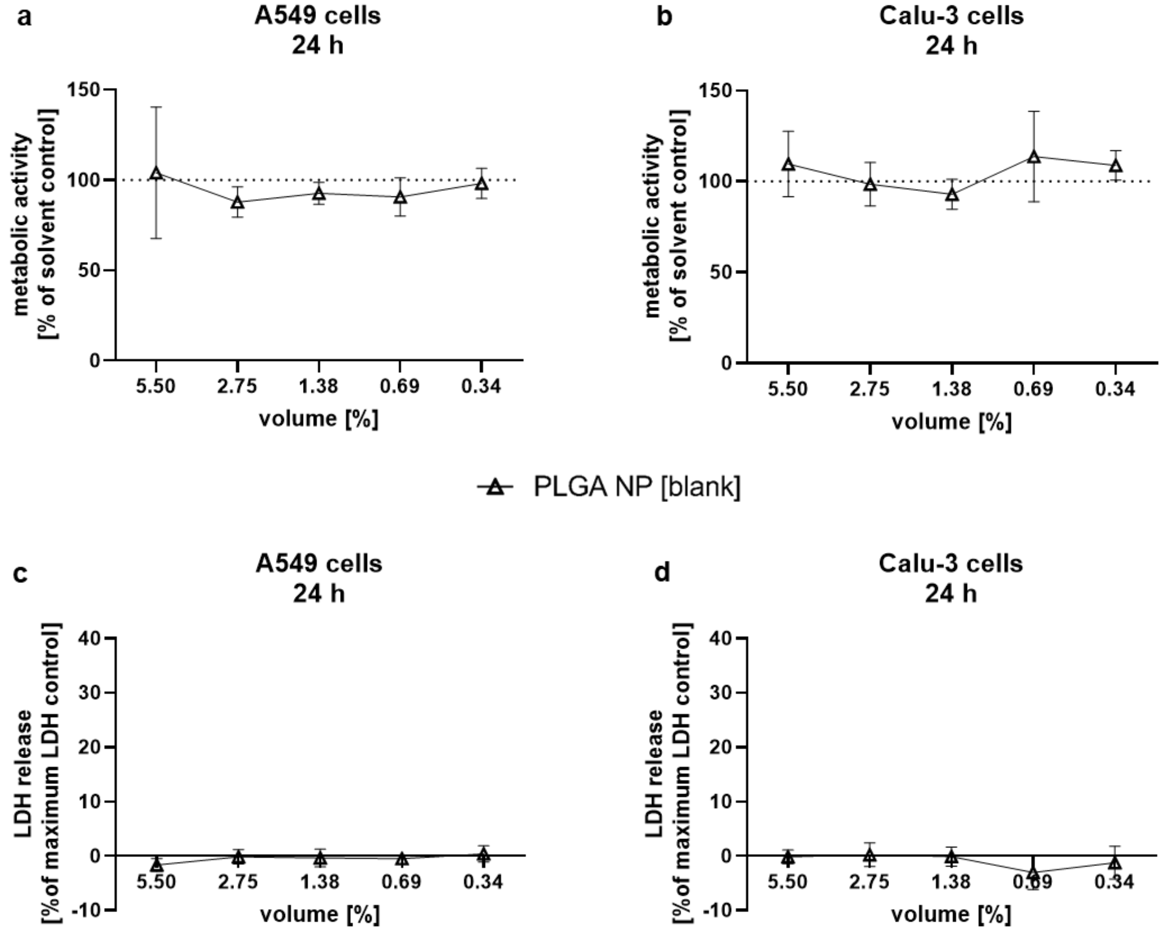

Supplementary Figure S1: PLGA NP [blank] has no impact on the cellular metabolic activity and LDH release. A549 (**a**,**c**) and Calu-3 (**b**,**d**) cells were treated with the indicated volumes of PLGA NP [blank], corresponding to the 6BIGOE concentrations shown in Figure 2a-d, for 24 h. (**a**,**b**) The metabolic activity was measured using MTT assay and normalized to the solvent control-treated samples (H_2O). The data represent the mean ± SD of four independent experiments with two technical replicates. Statistical significance was determined by two-way ANOVA with Dunnett's multiple comparisons test. (**c**,**d**) LDH level was measured by using cell supernatants after 24 h treatment with PLGA NP [blank]. The lysis buffer-treated sample served as the maximum LDH release control and was arbitrarily set to 100%, medium-treated samples were used as spontaneous LDH release control and represent 0%. The data represent the mean ± SD of three independent experiments with two technical replicates. Values are given in percent of the maximum LDH control. Statistical significance was determined by two-way ANOVA with Dunnett's multiple comparisons test.

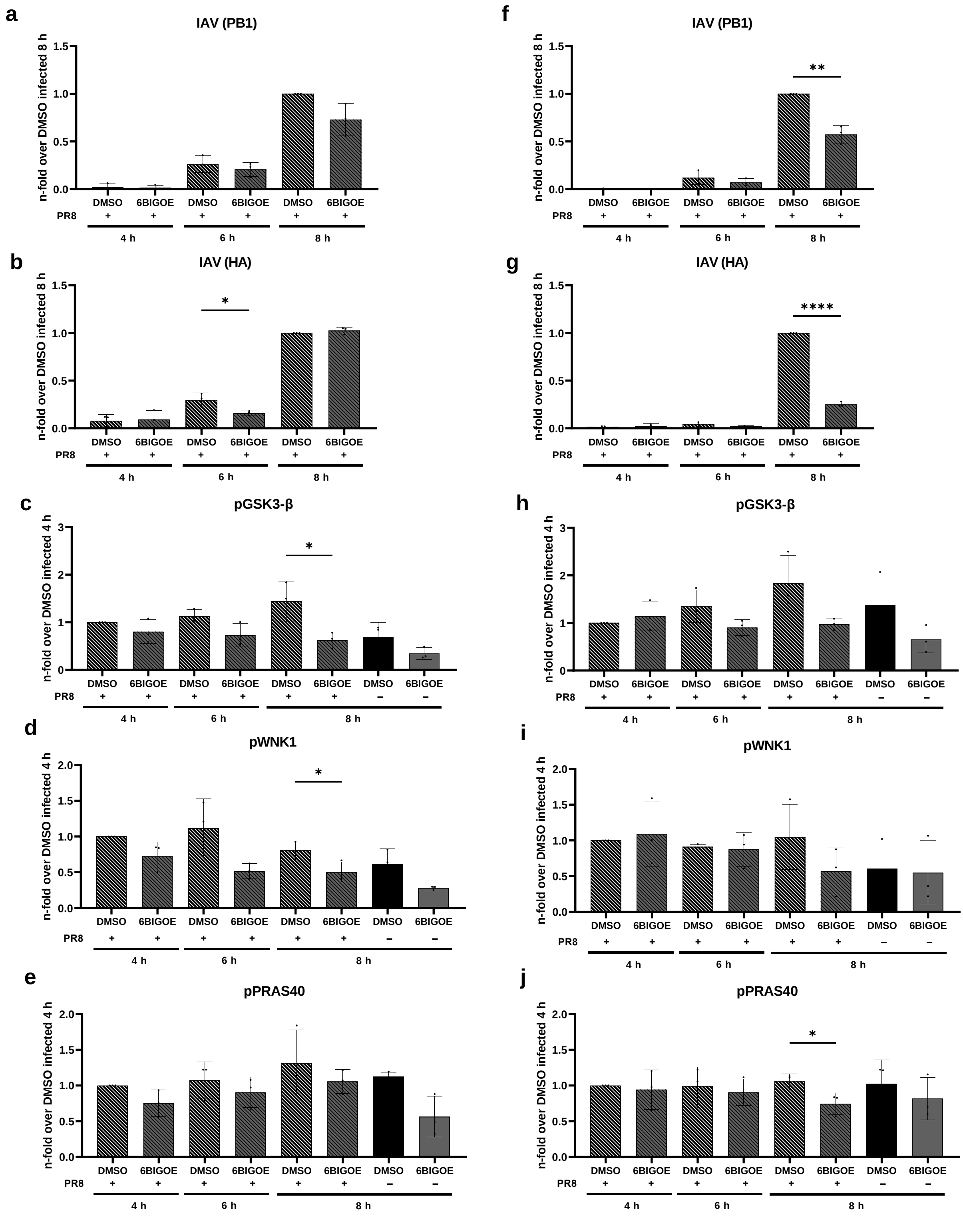

Supplementary Figure S2: 6BIGOE treatment causes a reduction of viral protein and signalling pathways. A549 (**a**-**e**) and Calu-3 (**f**-**j**) cells were infected with PR8 (5 MOI (**a**-**e**) or 1 MOI (**f**-**j**)) for 30 min and were subsequently left untreated or treated with 0.5 μM (**a**-**e**) or 1 μM (**f**-**j**) of 6BIGOE. Cell lysates were harvested after 4 h, 6 h and 8 h p.i. for Western blot analysis. Effect of 6BIGOE on viral HA and PB1, as well as PRAS40, WNK1 and GSK-3β phosphorylation is presented as n-fold over 8 h DMSO infected for HA, PB1 or n-fold over 4 h DMSO infected for pPRAS40, pWNK1 and pGSK-3β. Quantification of three independent experiments is depicted. Statistical significance was assessed using an unpaired t-test to compare 6BIGOE-treated (+) samples with solvent-treated (–) samples at each time point. * $p < 0.05$; ** $p < 0.01$; **** $p < 0.0001$.
